# Isolated perianal fistulizing Crohn’s disease in contemporary clinical practice: a multicenter ECCO-CONFER cohort study

**DOI:** 10.1093/ecco-jcc/jjag130

**Published:** 2026-09-11

**Authors:** Kumarini Basnayake, Deloshaan Subhaharan, David Drobne, Parakkal Deepak, Sami Samaan, Alessia La Mantia, Olga Maria Nardone, Justin R Howard, Francisco López Romero-Salazar, Pablo Ramírez Alcázar, Andrea Centritto, Bram Verstockt, Catherine Reenaers, Luke Hanna, Simone Saibeni, Rafal Filip, Melissa Hale, Daniela Pugliese, Edoardo Vincenzo Savarino, Davide Giuseppe Ribaldone, Jeroen Geldof, Magdalena Gawron-Kiszka, Cristina Bezzio, Anna Gojdičová, Edyta Zagórowicz, Aranzazu Jauregui-Amezaga, Thilini Basnayake, Stefano Festa, James L Alexander, Rachel Cooney, Tim Raine

**Affiliations:** Department of Gastroenterology, Cambridge University Hospitals NHS Foundation Trust, Cambridge, United Kingdom; Department of Gastroenterology, Cambridge University Hospitals NHS Foundation Trust, Cambridge, United Kingdom; Department of Gastroenterology, University Medical Centre, Ljubljana, Slovenia; Medical Faculty, University of Ljubljana, Ljubljana, Slovenia; Division of Gastroenterology, Washington University School of Medicine in Saint Louis, St Louis, MO, United States; Division of Gastroenterology, Washington University School of Medicine in Saint Louis, St Louis, MO, United States; Digestive and Interventional Endoscopy Unit, ASST Grande Ospedale Metropolitano Niguarda, Milan, Italy; Department of Public Health, University Federico II of Naples, Naples, Italy; Division of Gastroenterology Hepatology and Nutrition, University of Minnesota, Minneapolis, MN, United States; Department of Gastroenterology, Hospital Universitario 12 de Octubre, Madrid, Spain; Department of Gastroenterology, Hospital Universitario 12 de Octubre, Madrid, Spain; Department of Translational Medicine, University of Eastern Piedmont, Novara, Italy; Department of Gastroenterology and Hepatology, University Hospitals Leuven, Leuven, Belgium; University Hospital (CHU) & GIGA Institute, Liege University, Liege, Belgium; IBD Unit, St Mark’s Hospital, London North West University Healthcare NHS Trust, London, United Kingdom; IBD Unit, Gastroenterology Unit, Rho Hospital, ASST Rhodense, Rho, Italy; Department of Gastroenterology with IBD Unit, St. Jadwiga Queen Hospital No. 2, Rzeszów, Poland; Department of Gastroenterology, Sheffield Teaching Hospitals NHS Foundation Trust, Sheffield, United Kingdom; Department of Translational Medicine and Surgery, Università Cattolica del Sacro Cuore, Rome, Italy; Department of Surgery Oncology and Gastroenterology, University of Padua, Padua, Italy; Department of Medical Sciences, University of Turin, Turin, Italy; Department of Gastroenterology and Hepatology, Ghent University Hospital, Ghent, Belgium; Department of Gastroenterology and Hepatology, Prof. K. Gibiński University Clinical Centre of Medical University of Silesia in Katowice, Katowice, Poland; IBD Center, IRCCS Humanitas Research Hospital, Milan, Italy; Gastroenterology Center, Poliklinika Bezručova, Bratislava, Slovakia; Department of Oncological Gastroenterology, Maria Sklodowska-Curie Memorial Cancer Centre and Institute of Oncology, Warsaw, Poland; Department of Gastroenterology and Hepatology, University Hospital Antwerp, Edegem, Belgium; Institute for Biomedicine and Glycomics, Griffith University, Queensland, Australia; IBD Unit, San Filippo Neri Hospital, Rome, Italy; IBD Unit, St Mark’s Hospital, London North West University Healthcare NHS Trust, London, United Kingdom; Department of Gastroenterology and Hepatology, Imperial College Healthcare NHS Trust, London, United Kingdom; Department of Gastroenterology, University Hospitals Birmingham NHS Foundation Trust, Birmingham, United Kingdom; Department of Gastroenterology, Cambridge University Hospitals NHS Foundation Trust, Cambridge, United Kingdom

**Keywords:** isolated perianal Crohn’s disease, perianal fistula, Crohn’s disease, natural history

## Abstract

**Background and Aims:**

Isolated perianal fistulizing Crohn’s disease (IPFCD) is an uncommon presentation of Crohn’s disease with an uncertain natural history. We aimed to characterize contemporary diagnostic strategies, management approaches, and long-term outcomes, including progression to luminal Crohn’s disease.

**Methods:**

We conducted a retrospective multicenter cohort study across 25 centers within the ECCO-CONFER network. Adult patients with IPFCD and no evidence of luminal Crohn’s disease at baseline were included. The primary outcome was progression to luminal Crohn’s disease. Secondary outcomes included clinical and radiological fistula response and patterns of medical and surgical management. Time-to-event analyses were performed using Kaplan–Meier methods.

**Results:**

A total of 121 patients were included with a median follow-up of 76 months (IQR 46-118). Diagnostic pathways were heterogeneous, with most patients undergoing colonoscopy (91.7%) and pelvic magnetic resonance imaging (63.6%), alongside variable use of additional modalities. Management was intensive, with 76.9% receiving biologic therapies and 85.1% undergoing combined medical–surgical treatment. Complete clinical remission was achieved in 57% of patients, while radiological healing was observed in 46.4%. During follow-up, 42 (34.7%) patients developed luminal Crohn’s disease. Median progression-free survival was 145.6 months, with a median time to luminal diagnosis of 110 months (IQR 40-152). No consistent predictors of progression were identified.

**Conclusions:**

Approximately one-third of patients with IPFCD developed luminal Crohn’s disease over long-term follow-up, typically after several years. However, many patients remain free of intestinal involvement, reflecting a heterogenous disease course and highlighting the need for standardized evaluation and improved risk stratification.

KEY MESSAGES **What is already known on this topic?** 

- Isolated perianal fistulizing Crohn’s disease is a recognized but poorly defined phenotype
- Prior studies are small and report highly variable rates of progression to luminal Crohn’s disease
- Diagnostic and treatment strategies remain inconsistent

**What this study adds?** 

- In this large multicenter cohort, approximately one-third of patients developed luminal Crohn’s disease during long-term follow-up
- Median time to progression exceeded 9 years, highlighting delayed disease evolution
- Diagnostic pathways were highly heterogeneous, with frequent use of multimodal investigations
- Despite intensive treatment, clinical and radiological outcomes remain modest and discordant
- No consistent clinical predictors of progression were identified

**How this study might affect research, practice or policy?** 

- Patients with isolated perianal fistulizing Crohn’s disease require structured baseline evaluation and ongoing long-term surveillance
- A multimodal diagnostic approach may improve detection of subclinical luminal disease
- Therapeutic decision-making remains challenging in the absence of clear predictors
- Standardized diagnostic frameworks and prospective studies are needed to guide management

## 1. Introduction

Isolated perianal fistulizing Crohn’s disease (IPFCD), defined by the presence of perianal fistulae in the absence of current or prior luminal Crohn’s disease, represents an uncommon but clinically important presentation of inflammatory bowel disease (IBD).^1^^,^^2^ In contrast to typical Crohn’s disease, which is characterized by transmural inflammation of the gastrointestinal tract, IPFCD lacks endoscopic, histological, or radiological evidence of intestinal disease at diagnosis.^3^ This phenotype presents a significant diagnostic and therapeutic challenge, as it may closely mimic idiopathic cryptoglandular fistulae and hidradenitis suppurativa, with important implications for prognosis, surveillance, and treatment selection.^4^^,^^5^

The pathogenesis and clinical behavior of perianal fistulae differ substantially between Crohn’s-associated and cryptoglandular disease. In Crohn’s disease, fistula formation is driven by transmural inflammation, immune dysregulation, epithelial–mesenchymal transition, and impaired mucosal healing, whereas cryptoglandular fistulae arise from localized infection of the anal glands without systemic inflammatory disease.^6^ Clinically, Crohn’s-associated fistulae are more likely to exhibit complex features, including multiple tracts, horseshoe extensions, supra-levator involvement, and recurrent sepsis.^7^ These characteristics are associated with reduced response to conventional surgical approaches alone and typically necessitate a combined medical and surgical strategy within a multidisciplinary framework.^7–9^ The introduction of anti-tumor necrosis factor (TNF) therapy has transformed the management of fistulizing Crohn’s disease, demonstrating improved rates of fistula closure and sustained remission in landmark clinical trials.^10^^,^^11^ However, a substantial proportion of patients do not achieve or maintain response, and significant unmet clinical need remains. Contemporary evidence further supports combined surgical and medical strategies over surgical management alone in complex perianal Crohn’s disease.^12^

The diagnosis of IPFCD remains challenging and is based largely on exclusion of luminal Crohn’s disease using a combination of pelvic magnetic resonance imaging (MRI), examination under anesthesia (EUA), histopathological assessment, and variable use of luminal investigations.^4^ However, there is substantial heterogeneity in diagnostic pathways, and until recently, no standardized definition of isolated perianal Crohn’s disease has been established.^13^^,^^14^ Existing studies are limited by small sample sizes and variability in the extent of luminal assessment, reflecting differences in the availability and utilization of contemporary modalities such as video capsule endoscopy (VCE), intestinal ultrasound (IUS), magnetic resonance enterography (MRE), and computed tomography enterography (CTE).^14–17^ Consequently, the natural history of IPFCD remains poorly defined, which may also reflect challenges in recognizing Crohn’s disease at initial presentation in patients presenting with perianal fistulae, particularly regarding the risk of subsequent luminal disease. The optimal threshold for initiating immunomodulatory or biologic therapy in the absence of intestinal involvement also remains uncertain.^14^^,^^17–21^

We report the largest multicenter study to date of IPFCD, incorporating analysis of contemporary diagnostic pathways, management strategies, and long-term outcomes in patients managed at expert IBD centers across Europe and North America.

## 2. Materials and methods

### 2.1. Study design and setting

We conducted a retrospective, multicenter cohort study of adult patients with IPFCD across 25 participating centers. Sites were identified through a structured international call for cases distributed to members and affiliated centers of the European Crohn’s and Colitis Organisation (ECCO). The study was conducted within the framework of the ECCO Collaborative Network for Exceptionally Rare (CONFER) cases, an initiative established by ECCO to facilitate the collection of rare and uncommon clinical presentations through international collaboration. Cases are identified through coordinated calls to ECCO members, allowing aggregation of similar cases that would otherwise remain limited to a small single-center series. Patients were identified through clinical databases and specialist IBD services at each participating site. Contributing centers obtained institutional approval where required.

### 2.2. Study population

Eligible patients were adults aged ≥18 years with a diagnosis of perianal fistula without evidence of luminal Crohn’s disease at baseline, defined as the time of initial diagnosis of a perianal fistula.

Inclusion required a diagnosis of Crohn’s-associated perianal fistula based on expert gastroenterology and colorectal surgical assessment, supported by a multidisciplinary evaluation incorporating findings from EUA and radiological imaging where available, rather than fistula complexity alone. All patients were required to have undergone at least one luminal investigation within 12 months of baseline demonstrating no evidence of luminal Crohn’s disease. Acceptable modalities included colonoscopy, MRE, CTE, VCE, or IUS. Patients with established or concurrent luminal Crohn’s disease at baseline were excluded. To minimize misclassification of early luminal disease, patients who developed objective evidence of luminal Crohn’s disease within 12 months of baseline were also excluded from the final IPFCD cohort.

### 2.3. Data collection

Data extraction was performed locally by investigators at each participating center. Data were entered into a standardized electronic case report forms hosted on the REDCap platform and consolidated into a multicenter dataset for analysis. Owing to the retrospective multicenter design, formal source data verification was not undertaken. Data completeness varied across variables All data were anonymized prior to analysis and handled in accordance with local regulatory requirements.

Electronic medical records were reviewed at each participating center to extract demographic and clinical variables including age, sex, ethnicity, smoking status, relevant comorbidities, and family history of IBD. Disease-related variables included fistula characteristics (complexity, classification type, presence of abscess or collections, prior perianal interventions, and histology where available), as well as details of baseline diagnostic assessment. Information on diagnostic investigations was collected and categorized into perianal and luminal modalities. Perianal assessments included EUA and pelvic MRI, while luminal evaluation included colonoscopy, MRE, CTE, VCE, and IUS. The frequency, type, and number of investigations performed per patient were recorded. Treatment-related data were collected, including medical therapies (antibiotics, immunomodulators, and biologics) and surgical interventions (seton placement, drainage procedures, and fistulotomy), along with timing of therapy initiation relative to diagnosis. Where available, follow-up data were extracted to assess clinical and radiological outcomes, including response to therapy and disease course over time.

### 2.4. Definitions

The following definitions are aligned with established criteria used in prior interventional trials of perianal Crohn’s disease.^22^

Fistula outcomes were categorized as clinical remission, partial response, or persistently active disease based on documented symptoms and fistula drainage.

Complete clinical remission was defined as closure of all treated external openings that were draining at baseline, confirmed by the absence of drainage on gentle compression. Partial clinical response was defined as closure of ≥50% of treated external openings.

Radiological response was assessed using follow-up MRI, where available, and categorized as: (1) complete resolution or residual fibrosis/sinus only; (2) persistent tract(s) without associated collection >2 cm; and (3) persistent tract(s) with associated collection >2 cm.

### 2.5. Progression to luminal Crohn’s disease

Progression was defined as the development of objective evidence of luminal Crohn’s disease on endoscopic, histological, or radiological assessment during follow-up. Time to progression was calculated from baseline, defined as the time of initial diagnosis of a perianal fistula, to the first documented evidence of luminal disease.

### 2.6. Outcomes

The primary outcome was progression to luminal Crohn’s disease during follow-up. Secondary outcomes included clinical and radiological fistula response at last follow-up, as well as the utilization and patterns of medical and surgical therapies.

### 2.7. Statistical analysis

Descriptive statistics were used to summarize baseline characteristics, diagnostic investigations, management strategies, and clinical outcomes. Continuous variables were reported as mean (standard deviation [SD]) or median (interquartile range [IQR]), as appropriate, and categorical variables as frequencies and percentages. Time-to-event analyses for progression to luminal Crohn’s disease were performed using Kaplan–Meier methods, with comparisons assessed using the log-rank test. Exploratory analyses were performed to evaluate factors associated with progression to luminal disease using univariable time-to-event analyses. All statistical tests were two-sided, with a *P*-value <.05 considered statistically significant. Analyses were performed using GraphPad Prism (GraphPad Software, San Diego, CA, USA).

## 3. Results

### 3.1. Baseline characteristics

A total of 121 patients with IPFCD were included across 25 centers. The median duration of follow-up was 76 months (IQR 46-118 months). The cohort comprised 53 (43.8%) female patients, with 77 (63.6%) patients aged <40 years. The majority of patients were nonsmokers (63.6%) and of white ethnicity (82.5%), while 15.8% had a family history of IBD. Most patients had complex fistulae (86.8%), with transsphincteric tracts being the most common Parks classification (52.9%). A baseline perianal abscess was present in 51.2% of cases. Patients had undergone a median of three (IQR 2-4) prior perianal surgical interventions, reflecting a high burden of refractory disease. Baseline characteristics are summarized in Table 1.

**Table 1.** Baseline characteristics of patients with isolated perianal fistulizing Crohn’s disease.

Characteristic	**Total participants (*n*** = **121)**
**Female, *n* (%)**	53 (43.8)
**Age at diagnosis of fistula, years (%)**	
** <30**	51 (42.1)
**30-39**	26 (21.5)
**≥40**	44 (36.4)
**Smoking status, *n* (%)**	
** Never smoker**	77 (63.6)
**Current smoker**	24 (19.8)
**Ex-smoker**	14 (11.6)
**Unknown**	6 (5.0)
**Ethnicity, *n* (%)**	
** White**	99 (82.5)
**Asian**	11 (9.2)
**Arab**	4 (3.3)
** Black/African/Caribbean**	3 (2.5)
** Other**	2 (1.7)
**Mixed ethnic group**	1 (0.8)
**Unknown**	1 (0.8)
**Family history of IBD, *n* (%)**	
** No**	95 (79.2)
**Yes**	19 (15.8)
**Unknown**	7 (5.8)
**Fistula complexity, *n* (%)**	
**Simple**	16 (13.2)
**Complex**	105 (86.8)
**Parks classification, *n* (%)**	
**Transsphincteric**	64 (52.9)
**Intersphincteric**	33 (27.3)
**Extrasphincteric**	6 (5.0)
**Suprasphincteric**	5 (4.1)
**Unknown**	13 (10.7)
**Presence of baseline perianal abscess, *n* (%)**	62 (51.2)
**Median number of prior perianal surgeries, *n* (IQR)**	3 (2-4)

IBD, inflammatory bowel disease; IQR, interquartile range.

### 3.2. Baseline diagnostic pathways

**Figure 1.**
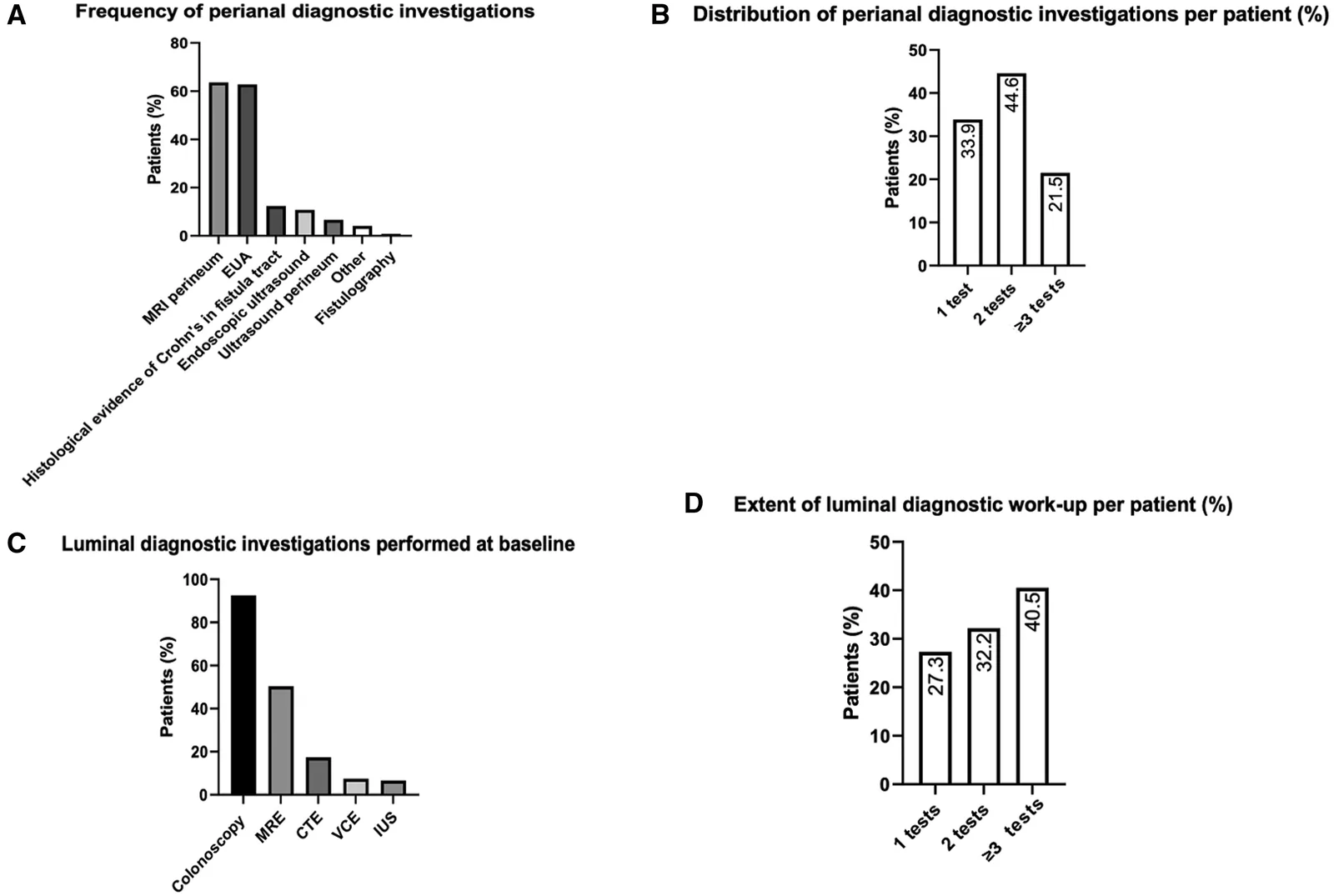
Diagnostic evaluation at baseline in patients with IPFCD. (A) Frequency of perianal diagnostic investigations performed at baseline. (B) Number of perianal diagnostic investigations per patient at baseline. (C) Frequency of luminal diagnostic investigations performed at baseline to exclude concurrent luminal Crohn’s disease. (D) Extent of luminal diagnostic work-up at baseline.

Substantial heterogeneity was observed in baseline diagnostic evaluation across centers (Figure 1).

Perianal assessment was most commonly performed using pelvic MRI in 77 (63.6%) patients, and EUA in 76 (62.8%) patients (Figure 1A). Overall, patients underwent a median of two perianal investigations (IQR 1-2) at baseline, with a mean of 1.62 investigations per patient (Figure 1B). A small proportion of patients (13.2%) did not undergo either MRI or EUA at baseline; in these cases, the diagnosis of perianal fistula was established clinically by colorectal specialists based on characteristic examination findings and/or operative documentation, reflecting real-world variability in practice.

All patients underwent at least one luminal investigation within 12 months of baseline to exclude concurrent Crohn’s disease (Figure 1C). Colonoscopy was performed in 111 of 121 (91.7%) patients. Among those undergoing colonoscopy, ileal intubation was achieved in 91 of 111 (82.0%) cases. In patients without ileal intubation, additional small bowel imaging (MRE, CTE, IUS, or VCE) was undertaken in 13 (65.0%) patients. Cross-sectional imaging was widely utilized, with MRE performed in 50% of patients and CTE in 17%. Patients underwent a median of two luminal investigations (IQR 1-2), with a mean of 2.2 investigations per patient. Notably, 40% of patients underwent three or more luminal investigations, reflecting a multimodal approach to diagnostic evaluation (Figure 1D).

### 3.3. Management strategies

Management approaches were heterogeneous and are summarized in Table 2. Medical therapy was widely utilized. Antibiotics were prescribed in 94 (77.7%) patients, immunomodulators in 62 (51.2%), and biologics in 93 (76.9%). Among those receiving biologics, infliximab was used in 70 (75.3%), adalimumab in 18 (19.4%), and ustekinumab in five (5.4%). One patient received mesenchymal stem cell therapy as part of advanced treatment. Surgical intervention was also common, with the majority of patients undergoing at least one perianal procedure, most frequently seton placement and/or incision and drainage of abscesses. Overall, combined medical and surgical management occurred in 103 (85.1%) patients, consistent with the complexity of disease in this cohort.

**Table 2.** Medical and surgical management strategies in patients with isolated perianal fistulizing Crohn’s disease.

Management strategy	***n*** = **121**
**Medical therapy, *n* (%)**	
** Antibiotics**	94 (77.7)
** Immunomodulators**	62 (51.2)
** Biologics**	93 (76.9)
**Surgical therapy, *n* (%)**	
** Seton placement**	85 (70.2)
** Fistulotomy**	50 (41.3)
** Incision and drainage of abscess**	62 (51.2)
** Fecal diversion**	13 (10.7)
**Combined medical and surgical therapy, *n* (%)**	103 (85.1)
**Mesenchymal stem cell therapy**	1 (0.8)

### 3.4. Clinical and radiologic outcomes

Despite intensive and multimodal treatment, clinical and radiological outcomes remained variable (Table 3). At last follow-up, 69 (57.0%) patients achieved complete clinical remission, while 36 (29.8%) demonstrated a partial clinical response. Persistently active disease was observed in the remaining 16 (13.2%) patients.

**Table 3.** Clinical and radiological outcomes at follow-up.

Clinical outcome, *n* (%)	*n* = 121
**Complete remission**	69 (57.0)
**Partial response**	36 (29.8)
**Persistently active disease**	16 (13.2)
**Radiological outcome, *n* (%)**	***n*** = **110**
**Complete healing**	51 (46.4)
**Active disease**	59 (53.6)

Radiological follow-up with pelvic MRI was available in 110 (90.9%) patients. MRI-defined healing was observed in 51 (46.4%) patients, while 59 (53.6%) demonstrated persistent radiological activity. Among the 69 patients achieving clinical remission, concordant radiological healing was observed in 51 (73.9%) patients, while 11 (15.9%) had persistent radiological disease activity. The median interval between baseline and last MRI assessment was comparable between patients with radiological healing (40 months [IQR 28-63]) and those with persistent disease (50 months [27-106]), suggesting that differences in healing rates were not attributable to variation in follow-up duration.

### 3.5. Impact of biological therapy on outcomes

Biologic exposure was not associated with improved clinical or radiological outcomes at last follow-up. Rates of inactive disease were similar between biologic-exposed and nonexposed patients (90% vs 89%, *P* = .73), and radiological healing on follow-up MRI did not differ significantly between groups (42% vs 56%, *P* = .25).

### 3.6. Progression to luminal Crohn’s disease

**Figure 2.**
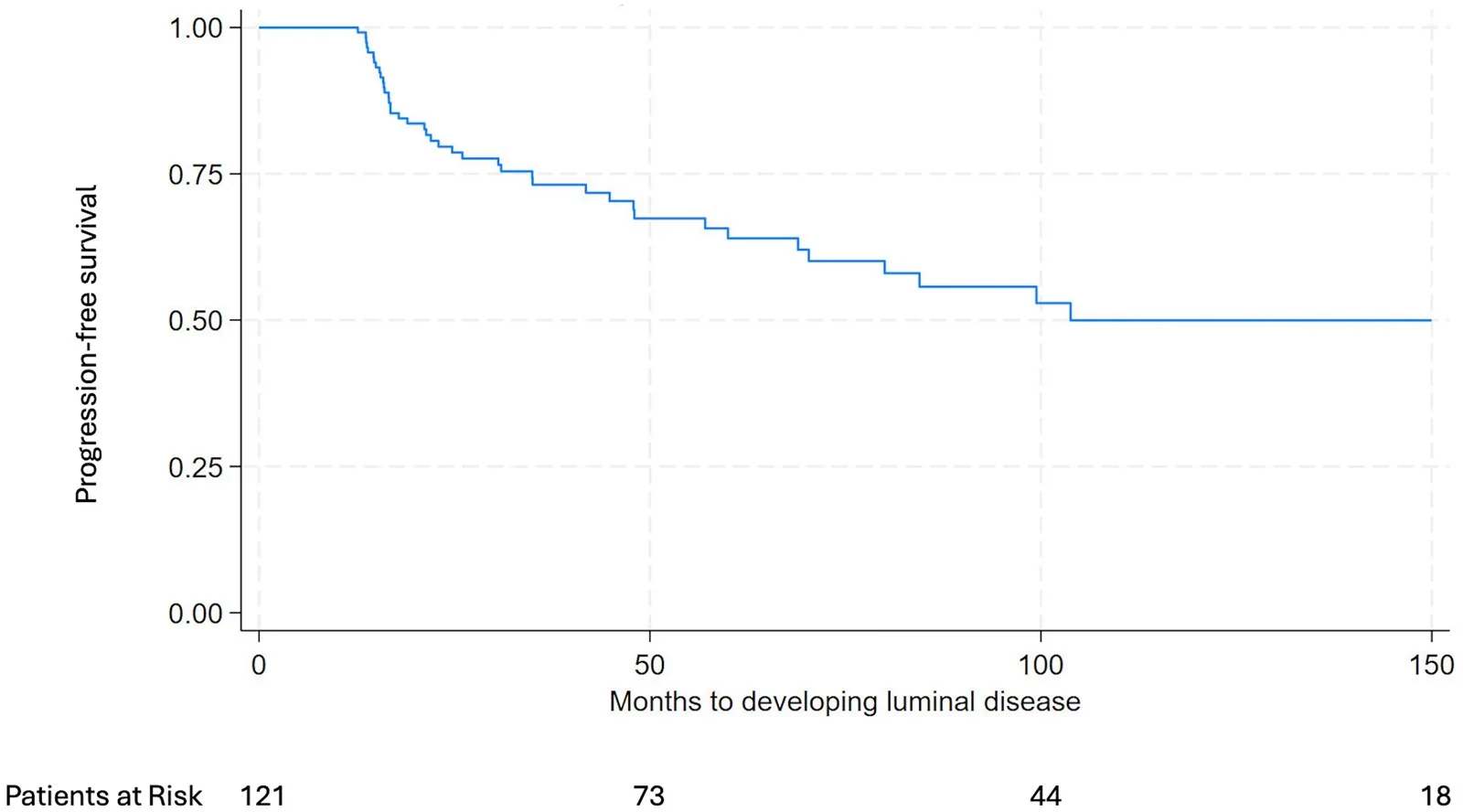
Progression-free survival from baseline to development of luminal Crohn’s disease. No progression events were observed within the first 12 months, consistent with study inclusion criteria.

During follow-up, 42 (34.7%) patients developed luminal Crohn’s disease, while 79 (65.3%) remained free of progression at their last follow-up (Figure 2). Kaplan–Meier analysis demonstrated a gradual decline in progression-free survival over time, with most progression events occurring within the first decade following initial perianal diagnosis. The estimated median progression-free survival was 145.6 months; however, relatively few patients remained under observation beyond 150 months, limiting the precision of estimates at later time points. Among patients who developed luminal disease, the median time to diagnosis was 110 months (IQR 40-152), indicating that progression may occur several years after the initial perianal presentation. A sensitivity analysis restricted to patients with successful terminal ileal intubation at baseline colonoscopy demonstrated a similar rate of progression to luminal Crohn’s disease, occurring in 31 of 91 (34.1%) patients compared with 42 of 121 (34.7%) patients in the overall cohort.

Among patients who developed luminal Crohn’s disease, disease location was most commonly ileocolonic or colonic, each observed in 17 of 42 (40.5%) patients, followed by isolated ileal disease in eight (19.0%), and upper gastrointestinal involvement in three (7.1%) patients. The majority of patients had nonstricturing, nonpenetrating disease (29/42, 69.0%), while 10 (23.8%) patients developed stricturing disease and three (7.1%) developed penetrating luminal disease.

Univariable Kaplan–Meier analyses were performed to explore factors associated with progression from IPFCD to luminal Crohn’s disease. Exposure to fistula-related surgical intervention during the disease course was associated with longer progression-free survival. Patients who had undergone one or more prior perianal surgery demonstrated longer progression-free survival compared with those who had not undergone surgery (*P* = .003). Fistula complexity was not associated with progression (*P* = .623). Similarly, early exposure to biologic therapy was not associated with progression-free survival (*P* = .355). No significant associations with progression were observed for sex, smoking status, family history of IBD, Parks classification, baseline perianal abscess, or age at diagnosis (*P* > .05).

## 4. Discussion

In this large multicenter ECCO-CONFER cohort of patients with IPFCD, approximately one-third of patients developed luminal Crohn’s disease during long-term follow-up. The risk of progression was greatest within the first decade following perianal disease onset, although a substantial proportion of patients remained free of luminal involvement even at extended follow-up. These findings highlight the heterogeneous nature of this phenotype and support the concept that IPFCD represents a dynamic stage within the broader spectrum of Crohn’s disease, rather than a fixed or uniform entity.

Data describing the natural history of IPFCD remain limited and are largely derived from small retrospective cohorts with variable definitions and follow-up.^1^^,^^14^^,^^23^ Reported progression rates have ranged widely from approximately 20% to over 50%, reflecting differences in baseline luminal exclusion, diagnostic intensity, and surveillance strategies.^14^^,^^24^^,^^25^ In this context, our study provides one of the most robust estimates to date of disease evolution through the use of structured baseline luminal evaluation and prolonged follow-up. The observed progression rate of approximately 35%, together with a median time to luminal diagnosis approaching a decade, underscores the prolonged period during which patients may remain classified as having isolated perianal disease despite an underlying predisposition to systemic involvement.

A key finding of this study is the marked heterogeneity in diagnostic pathways used to exclude luminal disease at baseline. While colonoscopy and pelvic MRI were commonly utilized, the use of additional modalities, including MRE, CTE, IUS, and VCE, varied substantially between centers. Many patients underwent multiple luminal investigations, reflecting both diagnostic uncertainty and the absence of standardized evaluation strategies, despite all patients undergoing at least one luminal investigation as required by study inclusion criteria. Emerging evidence suggests that reliance on a single modality may fail to detect subtle or early mucosal inflammation, supporting a multimodal approach to baseline assessment.^14^^,^^26^^,^^27^ The diagnostic modalities used reflected contemporary real-world practice across participating centers; however, the diagnostic performance of individual modalities for excluding subclinical luminal Crohn’s disease varies. Although a small number of patients were diagnosed during earlier eras when modalities such as pelvic MRI, VCE, and IUS were less widely available, the majority of the cohort was diagnosed in the contemporary era. Nevertheless, differences in access to and utilization of these investigations likely contributed to the heterogeneity observed in diagnostic pathways. In this context, structured frameworks such as the recently proposed TOPClass criteria may help standardize diagnostic evaluation, improve phenotypic precision, and facilitate comparability across studies.^28^

Despite widespread use of advanced medical therapies and combined medical–surgical management, clinical and radiological outcomes remained modest. Over three-quarters of patients received biologics, and the majority underwent repeated surgical interventions, reflecting a high treatment burden. However, complete clinical remission was achieved in only around half of patients, and radiological healing was observed in fewer than half of those undergoing follow-up imaging. Furthermore, discordance between clinical and radiological outcomes was evident, with persistent MRI activity observed in a subset of patients who were clinically quiescent. These findings highlight the limitations of symptom-based assessment and emphasize the importance of objective imaging in the evaluation of treatment response in perianal Crohn’s disease.^29^^,^^30^

Notably, biologic therapy was not associated with improved clinical, radiological, or progression outcomes in this cohort. This finding should be interpreted cautiously, as it is likely influenced by confounding by indication, with biologic therapy preferentially used in patients with more severe or refractory disease. Nonetheless, the absence of a clear signal for disease modification raises important questions regarding the role and timing of systemic therapy in patients without established luminal involvement and highlights the need for prospective studies evaluating treatment strategies in this specific population.

Exploratory analyses did not identify consistent predictors of progression to luminal Crohn’s disease. While prior perianal surgical intervention was associated with longer progression-free survival, this is unlikely to represent a causal effect and may instead reflect earlier specialist involvement or more intensive longitudinal monitoring. The absence of reliable clinical predictors underscores the unpredictability of disease evolution and highlights the need for improved risk stratification approaches, potentially incorporating molecular or imaging biomarkers.

From a clinical perspective, these findings support a strategy of comprehensive baseline evaluation to exclude luminal disease, followed by continued surveillance over time given the substantial risk of delayed progression. In our cohort, luminal disease at progression most commonly involved the colon and ileocolonic regions, and the majority of patients exhibited nonstricturing, nonpenetrating behavior at diagnosis. These findings suggest that surveillance strategies may need to prioritize colonic assessment, while also incorporating small bowel evaluation where clinically indicated. The optimal surveillance approach remains uncertain; however, integration ofnoninvasive biomarkers and periodic imaging may facilitate earlier detection of luminal involvement. Importantly, therapeutic decision-making in this population remains challenging, particularly in the absence of clear evidence to guide the initiation or escalation of systemic therapy.

This study has several strengths, including its multicenter international design, large sample size for a rare phenotype, detailed phenotyping, and prolonged follow-up duration. The use of standardized data collection through the ECCO-CONFER network enabled aggregation of cases that would otherwise be limited to small single-center series. However, several limitations should be acknowledged. The retrospective design introduces the potential for selection bias and variability in diagnostic and therapeutic approaches across centers. It also may have resulted in incomplete capture of some clinical variables due to missing data. Although not all patients underwent successful terminal ileal intubation at baseline colonoscopy, progression rates were similar in patients with ileal assessment and the overall cohort, supporting the robustness of the findings. In addition, the number of patients under observation at later time points was limited. Finally, the distinction between Crohn’s-associated and cryptoglandular fistulae relied in part on expert clinical judgment in the absence of universally accepted diagnostic criteria at the time of data collection.

In conclusion, approximately one-third of patients with IPFCD developed luminal Crohn’s disease over long-term follow-up, with progression occurring over a median of nearly a decade. However, many patients remain free of intestinal involvement, highlighting a heterogeneous disease course. The marked variability in diagnostic pathways, treatment responses, and absence of reliable predictors of progression emphasize the need for standardized evaluation strategies and improved risk stratification. Prospective studies incorporating structured diagnostic frameworks and biomarker-driven approaches are required to better define this complex and clinically challenging phenotype.

## Data Availability

The data supporting the findings of this study are available within the article and its supplementary materials.
